# Identifying the most productive breeding sites for malaria mosquitoes in The Gambia

**DOI:** 10.1186/1475-2875-8-62

**Published:** 2009-04-10

**Authors:** Ulrike Fillinger, Heleen Sombroek, Silas Majambere, Emiel van Loon, Willem Takken, Steven W Lindsay

**Affiliations:** 1 Disease Control & Vector Biology Unit, London School of Hygiene and Tropical Medicine, Keppel Street, London, WC1E 7HT, UK; 2 Laboratory of Entomology, Wageningen University, 6709 PD Wageningen, The Netherlands; 3 Institute for Biodiversity and Ecosystem Dynamics, University of Amsterdam, Nieuwe Achtergracht 166, 1018 WW Amsterdam, The Netherlands; 4 Ifakara Health Institute, PO Box 78373, Dar es Salaam, Tanzania

## Abstract

**Background:**

Ideally larval control activities should be targeted at sites that generate the most adult vectors, thereby reducing operational costs. Despite the plethora of potential mosquito breeding sites found in the floodplains of the Gambia River, about 150 km from its mouth, during the rainy season, only a small proportion are colonized by anophelines on any day. This study aimed to determine the characteristics of larval habitats most frequently and most densely populated by anopheline larvae and to estimate the numbers of adults produced in different habitats.

**Methods:**

A case-control design was used to identify characteristics of sites with or without mosquitoes. Sites were surveyed for their physical water properties and invertebrate fauna. The characteristics of 83 sites with anopheline larvae (cases) and 75 sites without (controls) were collected between June and November 2005. Weekly adult productivity was estimated with emergence traps in water-bodies commonly containing larvae.

**Results:**

The presence of anopheline larvae was associated with high invertebrate diversity (Odds Ratio, OR 11.69, 95% CI 5.61–24.34, p < 0.001), the presence of emergent vegetation (OR 2.83, 95% CI 1.35–5.95, p = 0.006), and algae (at borderline significance; OR 1.87, 95% CI 0.96–3.618, p = 0.065). The density of larvae was reduced in sites that were larger than 100 m in perimeter (OR 0.151; 95% CI 0.060–0.381, p < 0.001), where water was tidal (OR 0.232; 95% CI 0.101–0.533, p = 0.001), vegetation shaded over 25% of the habitat (OR 0.352; 95% CI 0.136–0.911, p = 0.031) and water conductivity was above 2,000 μS/cm (OR 0.458; 95% CI 0.220–0.990, p = 0.048). Pools produced the highest numbers of *Anopheles gambiae *adults compared with rice fields, floodwater areas close to the edge of the floodplain or close to the river, and stream fringes. Pools were characterized by high water temperature and turbidity, low conductivity, increased presence of algae, and absence of tidal water.

**Conclusion:**

There are few breeding sites that produce a high number of adult vectors in the middle reaches of the river in The Gambia, whereas those with low productivity are larger in area and can be found throughout the rainy season. Even though risk factors could be identified for the presence and density of larvae and productivity of habitats, the results indicate that anti-larval interventions in this area of The Gambia cannot be targeted in space or time during the rainy season.

## Background

Larval source management (LSM), a strategy which includes larviciding and source reduction (environmental manipulation, modification and elimination of aquatic habitats) for mosquito larval control, has long been used as a measure for malaria control in many parts of the tropics [1-7]. More recently larval control has been shown to be highly effective at reducing malaria transmission in Eritrea [8,9], Kenya [10,11] and Tanzania [12,13]. These studies were all carried out in sites with well-defined breeding sites that were not too extensive. However, it is not known whether this approach will be effective in areas where breeding sites cover large areas, as in the floodplains of large rivers or lakes. The work presented here is part of a series of studies recently undertaken in the The Gambia to determine whether larviciding with microbials [14] can reduce malaria transmission in an area of extensive seasonal flooding and to design rational application strategies for operational implementation.

In The Gambia, the landscape is dominated by the river and its floodplains dividing the small country into the river's north and south bank. Seasonal flooding creates large areas of water for extended periods of time and provides potential breeding sites for mosquitoes including the primary and secondary malaria vectors *Anopheles gambiae*, *Anopheles arabiensis*, *Anopheles melas, Anopheles funestus, Anopheles coustani *and *Anopheles pharaoensis *[15]. In The Gambia, malaria transmission is highly seasonal and peaks at the end of the rainy season which typically takes place from June to October [16,17]. In the middle reaches of the river in The Gambia (approximately 150 km from its mouths) there is considerable local variation in malaria intensity between villages. Malaria prevalence in children ranges between 5–90% [16,17] and the average entomological inoculation rate (EIR) between 0–150 infective bites per person per year [18].

The national strategy for malaria control in the country includes mosquito larval control [19], yet there has been no detailed evaluation of this methodology until now. The common malaria vectors in The Gambia are highly susceptible to commercial formulations of microbial larvicides in laboratory and field trials but the larvicides failed to exhibit any residual effect, which means that weekly applications are necessary to prevent adult mosquito emergence [14].

Mapping of all aquatic habitats in a study area of 400 km^2 ^was implemented under operational conditions [20] as a pre-requisite for successful anti-larval interventions [12,21] This was done to guide the larval control programme and determine whether specific habitat characteristics were associated with the presence of anopheline larvae [20]. It was hoped that any such characteristics could be used to guide interventions to target LSM at specific sites to reduce the logistics and costs necessary to implement larviciding at weekly intervals. The mapping revealed [20] a large number of shallow water bodies during the rainy season, primarily in the floodplains. The risk of finding anophelines increased in habitats located within the first one km stretch of the floodplains, from the landward edge towards the river. These were large in size and located in areas where grassy vegetation (including rice and sedge) dominated the land cover. Unfortunately, over 80% of all habitats shared similar features and these could not be used as criteria to help target anti-larval interventions. Thus, the type of water body and the habitat in which they were located could not be used for identifying sites for targeted LSM. Nevertheless, at any time of the year, only a small percentage of aquatic habitats actually contained mosquito larvae [20] suggesting that other criteria than those surveyed under operational conditions may be responsible for habitat colonization and productivity, such as microclimate, water quality and interactions with other aquatic organisms.

This experimental study was carried out to determine whether a detailed ecological and physical characterization of anopheline habitats could explain differences not only in vector colonization but also in larval density and, more importantly, densities of emerging adults in order to determine whether larval control can be targeted at particular sites [22].

## Methods

### Study area

The Gambia is a small, narrow country in West Africa whose borders mirror the meandering Gambia River. The country is less than 48 km wide, with a total area of 11,300 km^2^. The study was done on the north bank of the Gambia River, east of Farafenni town, around Balanghar Ker Nderry (UTM: 1510598N, 456756E) from June to November 2005 during the rainy season, the peak season of malaria transmission. Mosquito collections were carried out within an area of approximately 100 km^2 ^comprising the most common habitats found in the large river ecosystem [14,20]. The study area can be divided broadly into: (1) upland that is predominantly woodland savannah and farmland, where the main crops are millet and groundnuts, and (2) the river's floodplain, where large areas of alluvial soils are flooded during the rainy season and rice is grown.

### Habitat surveys

Our aim in the present study was to identify the characteristics of habitats with larvae, as well as those with the highest densities. Our surveys differed in a number of aspects from previously published work implemented under operational conditions [20]. Firstly, in this case, as with most larval surveys, sites were not selected randomly in space and time and are therefore subject to potential confounding. The case-control approach we adopted here allowed us to randomly select sites in space and time. Secondly, we used area samplers, a more accurate sampling tool for collecting larvae, than standard dippers when larval densities are low [14]. Thirdly, water chemistry and associated insect fauna was analyzed in association with larval abundance.

At the end of each week, records from the routine habitat surveillance were used to identify habitats that contained anopheline larvae in the last five days. This routine surveillance was implemented in preparation for a large-scale larviciding trial with the aim to map all available water bodies within the area every four to six weeks [20]. Each habitat's position was recorded with a handheld Global Positioning System (GPS, Garmin GPS 12 XL, 15 metres accuracy), received a unique habitat number and information on the presence and absence of mosquito larvae was noted. More details on the methodology have been described by Majambere and others [20]. Of all habitats surveyed the previous week, five sites colonized by *Anopheles *larvae and five sites where no *Anopheles *larvae were found, were selected randomly (irrespective of other habitat characteristics) without replacement using the web-based randomization tool [23]. These sites were surveyed intensively the following week as described below.

**Figure 1:**
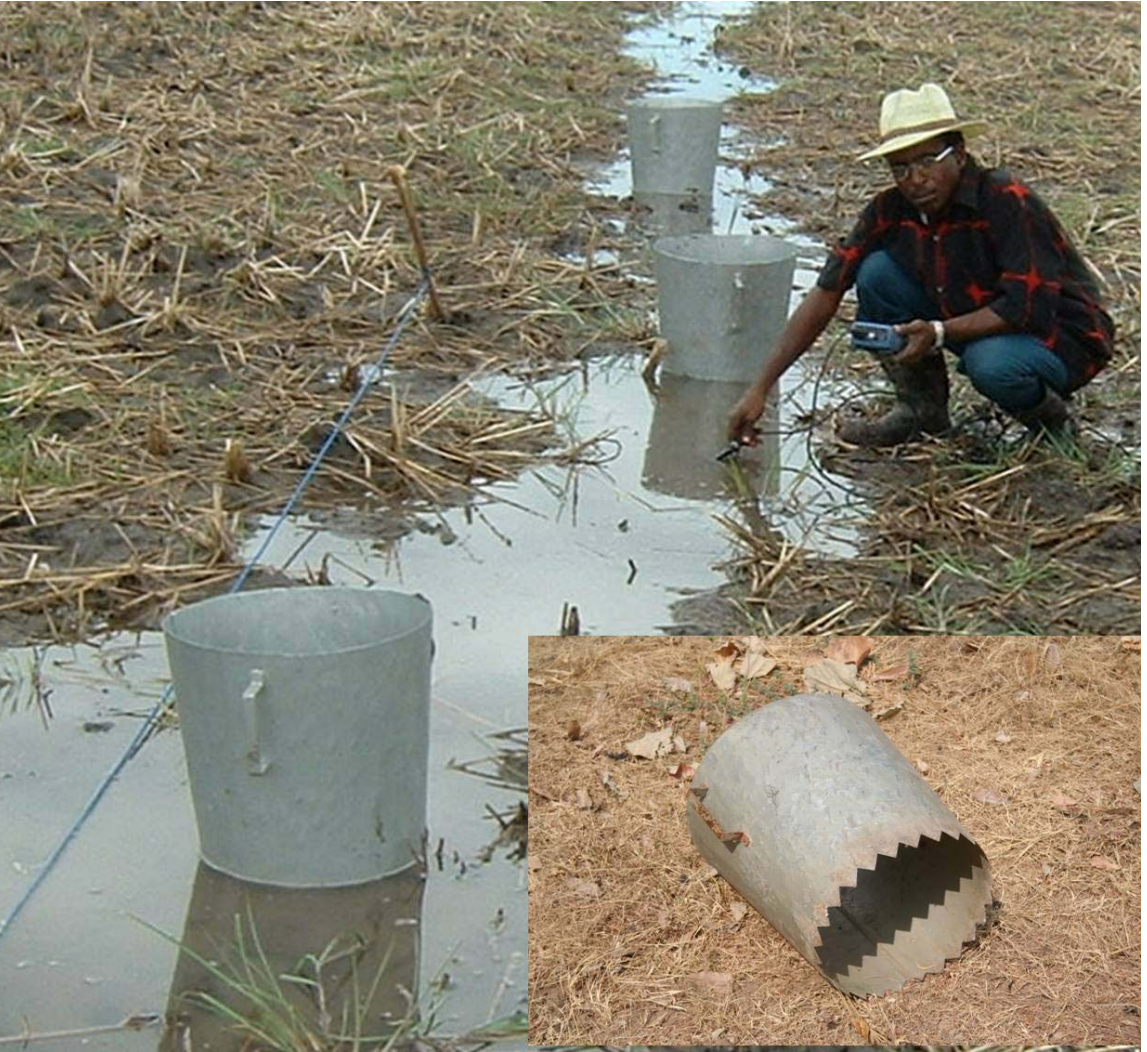
**Area samplers for sampling aquatic fauna**.

In each aquatic habitat, three samples of mosquitoes and other organisms present were taken with an area sampler (AS; Figure 1) [24] within 10 m of each other. Samples within each sampling site were pooled. The AS was a 39.5 cm long aluminium tube, with serrated teeth around the bottom lip to grip into the substrate. It had an upper diameter of 47 cm and a lower one of 40 cm sampling a surface area of 0.126 m^2^. The AS was plunged quickly into the water body in areas most likely to contain larvae (i.e. edge of water or near emergent vegetation [25]) and left for 30 seconds to allow the water to settle and larvae to come to the surface. A standard 350 ml dipper was used to empty the water from the AS and transfer it into a white plastic bowl containing clear water. Excess water was carefully removed to concentrate any organisms present in the bowl. All invertebrates were collected and placed in 98% ethanol before being transported to the laboratory for identification. All insects, excluding mosquitoes, were separated into the following taxonomic groups: beetle larvae (Coleoptera), bettle adults (Coleoptera), dragonfly and damselfly larvae (Odonata; sub-order Anisoptera and Zygoptera), mayfly larvae (Ephemeroptera), larvae of the Diptera families of non-biting midges (Chironomidae) and phantom midges (Chaoboridae) and water bugs (Heteroptera), which were further identified as broad-shouldered water striders (Veliidae), creeping water bugs (Noucoridae), greater water boatman (Notonectidae), lesser water boatman (Corixidae), pigmy backswimmers (Pleidae), pond skaters (Gerridae), water measurers (Hydrometridae) and water scorpion (Nepidae). Mosquito larvae were identified and counted as anopheline and culicine early (1^st ^and 2^nd ^stage larvae) and late instars (3^rd ^and 4^th ^stage larvae). Mosquitoes were identified on morphological characteristics, and members of the *An. gambiae *complex identified to species level by PCR analysis [26].

The presence of small fish and any vegetation present inside the AS was recorded. The presence of algae was confirmed by microscopic investigation of a water sample or the presence of filamentous algal mats. Each aquatic habitat was classified into one of the following categories (presenting the full diversity of aquatic habitats within the study area) which are usually found in succession from the village (upland) towards the main river (floodplains): (1) Brick or sand pits: borrow pits (>2 m diameter) resulting from brick-making or other construction activities, (2) Pools: discrete (<200 m diameter) and shallow (<50 cm) standing water bodies, usually drying out towards the end of the dry season, (3) Edges of floodwater: the shallow landward edges of the extensive floodwater in the floodplains of the river or its tributaries, usually associated with grass (*Paspalum *and *Sporobolus *sp.) and sedge (*Eleocharis *sp.), (4) Ponds: discrete and permanent water bodies, more than 100 m in circumference fed by groundwater and deeper than pools, (5) Water channels: used for irrigation or drainage, (6) Stream fringes: the shallow edges of permanent streams associated with grass or sedge, and tall reeds in deeper parts, (7) Puddles or tyre tracks: small natural or vehicle-made depressions, (8) Floodwater: inundated areas in the floodplain further away from the landward edge, towards the river, (9) Rice fields: seasonally flooded areas used to grow rice, and (10) Mangrove: water body characterized by densely growing mangrove trees (*Rhizophora *and *Avicennia *sp.) near the main river.

### Emergence trap collections

Data on mosquito adult emergence were collected in order to investigate the relative productivity of different habitats where larvae were frequently found. The extensive mapping of the study area in preparation for a large operational larval control trial showed that anopheline larvae occurred in a variety of habitats, even close to the river [20]. The baseline data also indicated that rice fields, floodwater areas with sedge and/or grass, stream fringes (water covering grassy edge) and pools were among the most commonly encountered aquatic habitats and were to a high proportion colonized by anopheline larvae [20]. The objective was to evaluate whether these habitats are equally productive in terms of adult emergence and to assess if distance to the river affects the larval development to adult stage. Here, the number of adults that emerged from any breeding site served as an indicator of risk of malaria transmission but other factors such as the size of emerging adults may also be important.

**Figure 2:**
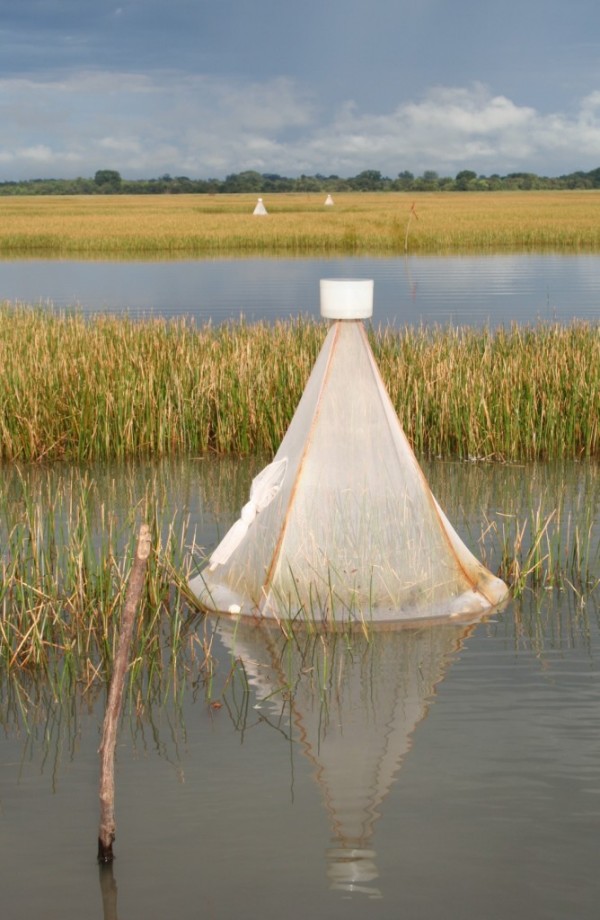
**Emergence traps used for estimating adult vector production per habitat type**.

Therefore, six areas were selected were the following habitats dominated respectively: (1) a collection of pools within the floodplain, (2) stream fringes within the floodplain, (3) rice fields at the edge of the floodplain, (4) rice fields close to the river, (5) floodwater areas (sedge/grass) at the edge of the floodplains, and (6) floodwater areas within the floodplains. These areas were chosen randomly from sites that contained *Anopheles *larvae at least once during the rainy season in 2004. Six floating emergence traps [27] were positioned in each of these habitats to sample adult insects continuously from June to November 2005 (Figure 2). These traps collected positively phototaxic arthropods that emerged from the water. Traps were positioned over water thought likely to contain anopheline larvae (e.g. at the edges of the habitat, over tuft of vegetation). Each trap was at least 50 m from its nearest neighbour. They were constructed from conical metal frames 1 m in height and 1 m in diameter (0.786 m^2 ^surface area) and covered in synthetic netting to reduce shading of the water, which might reduce invertebrate catches. Traps were made buoyant by attaching plastic 1 L bottles to the base with wire to allow the water to flow undisturbed under the trap, maintaining current, ambient temperature and oxygen, and other biotic and abiotic factors. Bottles were partly filled with sand to prevent the trap from being blown over by strong winds. Each trap was tethered with a length of rope to a wooden stake anchored to the ground allowing the trap to rotate freely. Traps placed over mature rice plants were not tethered.

The top of each cone opened into a plastic insect collection chamber (Bioform, Germany) with a transparent polystyrol lid. The chambers were filled with 250 ml of 60% glycol in order to kill and preserve flying insects that collected there. A netting sleeve on the side of each trap allowed flying insects caught within the netting cone to be removed with an aspirator. Traps were emptied weekly and specimens transported to the laboratory for identification. The dominant vegetation in and around the traps was recorded weekly as: (1) grass including rice (*Paspalum, Sporobolus *sp. or *Oryza sativa*), (2) sedge (*Eleocharis *sp.), (3) sea-purslane (*Sesuvium *sp.), (4) reeds (*Cyperus papyrus *and *Phragmites karka*), (5) algae (filamentous and single-celled), (6) floating plants e.g. *Azolla *sp., water-lilies or (7) no vegetation. After collection, the chambers and traps were cleaned of spiders and webs and other detritus which might lead to over- or underestimation of catch size. Traps were moved weekly to a new position within 10 m from the previous sampling point.

In the laboratory, the content of each collection chamber was filtered to remove the glycol and transferred to ethanol. Insects were classified into the following taxons: beetles (Coleoptera), dragon- and damselflies (Odonata); mayflies (Ephemeroptera), and water bugs (Corixidae, Hydrometridae, Nepidae, Notonectidae, or Noucoridae). Within the order Diptera the sub-order Brachycera were separated from the sub-order Nematocera. The latter were classified as phantom midges (Chaoboridae), non-biting midges (Chironomidae), blackflies (Simuliidae) or mosquitoes (Culicidae). Mosquitoes were identified and counted as anopheline and culicine males and females. All insects were identified with the aid of morphological keys and members of the *An. gambiae *complex identified by PCR analysis [26].

### Water physical measurements

Water measurements were made at each survey point. Water depth was measured with a measuring tape at three different locations within the sampling area and averaged. Whether the water was tidal or not was assessed visually. The overall perimeter size of the habitat was estimated as: (1) <10 m, (2) 10–100 m or (3) >100 m. In large areas of water it was also recorded whether the water outside the sampling area was deeper (>50 cm) than the area where samples were taken. The coverage of the water surface with tall emergent vegetation was visually estimated as: (1) <25%, (2) 25–50%, (3) 51–75%, and (4) >75%. Water conductivity, pH, temperature and dissolved oxygen content were measured with a multiparameter probe (350i WTW, Germany) and water turbidity with a turbidity meter (HANNA, USA). Measurements were made weekly at all larval survey points and in the areas of adult sampling. All samples were taken between 07:00 and 14:00 hrs.

### Statistical analysis

Proportions were compared using likelihood ratio chi-square analysis. Comparisons between means of normally distributed data were made using students t-test or one-way ANOVA in combination with a Gabriel post-hoc test. Means of data that could not be normalized were compared using the Kruskall-Wallis Test. The Bonferroni correction was used where applicable. The number of insect taxa was counted and the Shannon index (taking into account the number of identified taxa as listed above and their proportion in a sample) was used as a metric for species diversity [28]. Risk factor analyses were implemented using Generalized Estimating Equations (GEE) [29]. Variables were incorporated untransformed in the model and their impact on the presence or absence and on the density of anopheline larvae or adults tested. Binary data (presence or absence) was fitted to a binomial distribution with a logit link function. Count data were fitted to a negative binomial distribution with a log link function. GEE were also used to calculate mean *Anopheles *larval and adult densities, with site ID as subject units, log linked mosquito densities and habitat type as the factor. A stepwise backwards approach was used for the final models. Analyses were performed with SPSS version 15. Data from emergence traps that were not fully working were excluded from the analysis.

## Results

### Presence or absence of larvae

Between June and November 2005 data from 83 sites with anopheline larvae (cases) and 75 sites without (controls) were collected. Most sites were in the floodplains (81.6%) and a smaller sample in the uplands (18.4%). All major habitat types identified by Majambere and others [20] were represented in the random samples (Table 1).

**Table 1:** Habitat types surveyed for absence (controls) and presence (cases) of *Anopheles *larvae

	**Habitat type**	**Controls (n = 75)**	**Cases (n = 83)**
**Upland**	Puddles	2	3
	Man-made pits	3	4
	Pools	7	8
	Ricefields	0	2

**Floodplain**	Puddles	1	3
	Pools	6	7
	Stream fringes	7	15
	Ricefields	17	19
	Floodwater	32	22

A total of 5,784 invertebrates and 195 fish were collected from the 158 sites sampled. Culicid larvae were the most abundant organisms with 708 anopheline larvae (60% early and 40% late instars) and 3,868 culicine larvae (52% early and 48% late instars). Notably, only 15 anopheline pupae and 18 culicine pupae were caught in the area samplers. Among the predatory insects the lesser water boatman (n = 761) and greater water boatman (n = 187) were most abundant. Other water bugs (n = 20) included water measurers, pond skaters, water scorpions and creeping water bugs. In addition, 60 damselflies, 46 dragonflies, 32 beetle larvae and 51 beetle adults were collected. Only 4 chironomid larvae were collected in the area samplers probably because most species live within the sediment.

Comparison of cases and controls showed that the two groups were similar in most of their characteristics (Table 2). A similar proportion of habitats selected in both groups were tidal and deep and contained fish. The mean water depths, vegetation cover, water temperature at sampling time, pH, salinity, turbidity and oxygen saturation were also similar in the two groups. Only two characteristics were significantly different; anopheline larvae were found where there was high insect diversity and where water conductivity was low.

**Table 2:** Descriptive statistics for habitats without (controls) and with (cases) *Anopheles *larvae

	**Control**	**Cases**	**p**	χ^2^**/t**
PROPORTION*				
of tidal water bodies	72.0%	74.7%	0.701	0.147
of deep waterbodies (>50 cm)	25.3%	22.9%	0.720	0.129
of habitats with fish	20.0%	14.5%	0.355	0.854
of habitats with Culicinae	20.0%	53.0%	**<0.001**	18.351
of habitats with Odonata	14.7%	38.6%	**0.001**	11.349
of habitats with Coleoptera larvae	12.0%	27.7%	**0.014**	6.021
of habitats with Coleoptera adults	24.0%	39.8%	**0.034**	4.476
of habitats with Ephemeropera	5.4%	20.5%	**0.005**	7.845
of habitats with Heteroptera	26.7%	49.4%	**0.003**	8.589
MEAN (95% CI)**				
Diversity index	0.27 (0.18–0.35)	0.85 (0.73–0.97)	**<0.001**	7.898
Water depths (cm)	7.2 (5.5–8.9)	7.0 (5.6–8.5)	0.871	0.163
Vegetation cover of habitats (%)	50.3 (42.4–58.3)	48.6 (41.7–55.5)	0.745	0.326
Water temperature (°C)	30.4 (29.6–31.3)	29.4 (28.5–30.3)	0.111	1.044
pH	6.4 (6.2–6.6)	6.5 (6.3–6.7)	0.373	-0.894
Conductivity (μS/cm)	7009 (4700–9319)	3621 (2331–4911)	**0.010**	2.609
Turbidity (ntu)	136.9 (86.6–187.3)	191.4(128.4–254.4)	0.188	-1.323
Oxygen saturation (%)	72.4 (63.1–81.8)	83.2 (72.5–93.8)	0.133	-1.509

*Proportions are compared using χ^2^-test

**Means are compared using t-test

Stepwise backwards binary logistic regression (entered at first step: habitat type, habitat size, habitat depth, water body depth, percentage vegetation cover, presence of tidal water, type of emergent vegetation and presence of algae) revealed that the presence of *Anopheles *larvae was only associated with the presence of emergent vegetation (Odds Ratio, OR = 2.83, 95% Confidence intervals, CI 1.35–5.95, p = 0.006) and algae (OR 1.87, 95% CI 0.96–3.618, p = 0.065); however the latter only approached statistical significance. Similarly, the diversity of other organisms increased in the habitat when emergent vegetation (OR 1.88; 95% CI 1.23–2.89, p = 0.004) and algae (OR 1.87, 95% CI 1.40–2.49, p < 0.001) were present. Consequently, there was a highly significant association between the presence of *Anopheles *larvae and the diversity index (OR 11.69, 95% CI 5.61–24.34, p < 0.001). The impact of increasing conductivity on biological outcome measures was not clear-cut. Regression analyses did not reveal any significant associations between groups of increasing conductivity and the presence of *Anopheles *larvae. This might be due to the fact that the relationship between the variables was not linear.

There was only a very weak correlation between the presence of early and late instar larvae (r^2 ^= 0.095; p = 0.005). Early instars were found in 88% (73/83) of cases whilst late instars were only recorded in 59% (49/83). Both stages together occurred in only 47% of all cases (39/83). Therefore, binary analyses for both sub-groups were run separately. When modelling the risk factors associated with the presence of early instar anophelines in a stepwise backwards approach the likelihood of finding early instars was significantly higher when samples were taken in areas where the water was less than 10 cm deep (OR 11.00, 95% CI 1.23–98.60, p = 0.032) and in water-bodies that contained emergent vegetation (OR 2.86, 95% CI 1.32–6.20, p = 0.008) and algae (OR 2.36, 95% CI 1.18–4.72, p = 0.015). For late instars only the presence of vegetation (OR 2.07, 95% CI 0.90–4.76, p = 0.089) and algae (OR 1.92, 95% CI 0.96–3.83, p = 0.065) remained in the final model. Both factors only approached significance.

Of 708 anopheline larvae sampled, 166 larvae were identified to species level. *An. arabiensis *accounted for 37.3% of anophelines, *An. gambiae s.s. *for 4.8%, *An. melas *for 4.2% (total of *An gambiae s.l*. 46.3%), *An. coustani s.l. *for 31.9% (this includes *An. coustani *and *Anopheles ziemanni*, two species which can not be morphologically distinguished in the larval stage and were therefore grouped together throughout and referred to as *An. coustani s.l. *hereafter), *An. pharaoensis *for 11.4% and the remaining 10.2% were other anopheline species. Anopheline larvae were found in all habitat types surveyed. Whereas floodwater areas, rice fields and stream fringes were dominated by *An. coustani s.l. *and to a lesser extent by *An. pharaoensis*, pools, puddles and man-made pits were predominantly colonized by *An. gambiae s.l.*. *An. arabiensis *was the most common member of the *An. gambiae *complex and was the only species found in rice fields (Table 3).

**Table 3:** *Anopheles *larvae species composition per habitat type

**Habitat type**	**Total number of** **larvae sampled**	**Proportion of larvae** **identified in % (n)**	***Anopheles *species composition based on identified specimen in %**						
			
			***gambiae s.l.***	gambiae s.s.*	arabiensis*	melas*	***coustani***	***pharaoensis***	**others**
Floodwater (n = 22)	95	34.7 (33)	21.2	6.1	0.0	15.2	30.3	18.2	30.3
Rice field (n = 21)	71	32.4 (23)	17.4	0.0	17.4	0.0	47.8	8.7	26.1
Stream fringe (n = 15)	76	39.5 (30)	10.0	6.7	3.3	0.0	53.3	20.0	16.7
Pool (n = 15)	213	21.1 (45)	53.3	6.7	46.7	0.0	28.9	11.1	6.7
Puddle (n = 6)	158	20.3 (32)	87.5	3.1	81.3	3.1	6.3	0.0	6.3
Man-made pits (n = 4)	95	16.8 (16)	87.5	6.3	75.0	6.3	0.0	0.0	12.5

*species of the *An. gambiae *complex summarized under *An. gambiae *s.l.

### Larval density

**Figure 3:**
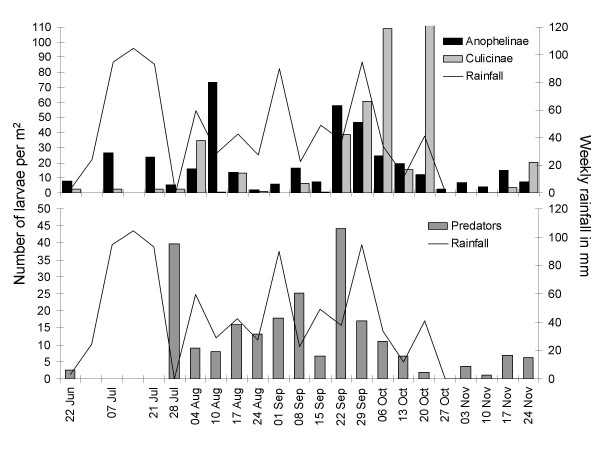
**Weekly average density of mosquito larvae and predatory insects per m^2 ^in relation to rainfall pattern**.

The mean number of *Anopheles *larvae per m^2 ^(considering cases only) was 22.6 (95% CI 11.6–33.5) during the study; early instars accounted for 60% of all larvae (13.5, 95% CI 6.7–20.3) and late instars for 40% (9.1, 95% CI 1.9–16.2). At the beginning of the rainy season in June/July mosquito larval densities were low and predatory invertebrates (e.g. beetles, dragon-and damselflies, water bugs) largely absent. *Anopheles *densities per m^2 ^were highest at the beginning of August and the end of September. Culicine mosquitoes were most abundant at the end of the rainy season (Figure 3).

Larval densities for each habitat type in the upland and floodplains of the River Gambia were highly variable (Table 4). Upland sites had a greater larval density (OR 2.70, 95% CI 1.05–6.98, p = 0.04), but adjusting for habitat type, this association was not significant indicating that high larval density is dependent on the habitat type or more importantly the characteristics associated with this habitat type, but not location. High larval densities were found in puddles (n = 3) and man-made pits in the upland (n = 4) and pools in the floodplains (n = 7). Floodplain pools had not only significantly higher larval densities than upland pools (n = 8) but all other habitat types sampled in the floodplain (Table 4).

**Table 4:** Mean (95% CI) *Anopheles *larval densities in habitats upland and in the floodplain

	**Upland**	**Floodplain**	p*
**Ricefields**	10.0 (3.3–30.3)^b^	2.7 (1.9–3.7)^b^	0.239
**Pools**	3.1 (2.0–4.8)^b^	26.9 (8.2–87.6)^a^	0.027
**Puddles**	47.7 (29.5–77.1)^b^	5.00 (2.6–9.7)^b^	0.050
**Man-made pits**	23.8 (5.4–104.8)^b^	-	n/a
**Floodwater**	-	4.3 (3.1–6.1)^b^	n/a
**Stream fringes**	-	5.1 (3.0–8.6)^b^	n/a
p*	0.066	0.027	

a, b: different letters in lines (p < 0.05) or rows (p < 0.0125) indicate significance (in floodplain only comparison between pools and other habitats was run)

*Kruskall-Wallis-Test

Whilst the presence or absence of anopheline larvae was associated with the presence of vegetation or algae, the density of larvae was dependent on a variety of physical factors. Larval density decreased with: (1) increasing size of habitats, (2) increasing vegetation cover shading the habitat, (3) the presence of tidal water and (4) when the water body as a whole was on average deeper than 50 cm (Table 5). There was no difference for early and late instars. The water depth, presence of vegetation or algae at the sampling point, the presence or abundance of fish, or the increase in the diversity index were not significantly associated with anopheline larval density, neither with early nor late instars nor any specific *Anopheles *species.

**Table 5:** Univariate analyses of factors associated with *Anopheles *larval density

	**Odds ratio**	**95% CI**		**p**
**Habitat perimeter**				
1–10 m	1			
10–100 m	0.434	0.096	1.961	0.278
>100 m	0.151	0.060	0.381	<0.001

**Vegetation coverage**				
0–25%	1			
25–50%	0.352	0.136	0.911	0.031
50–75%	0.259	0.105	0.642	0.004
75–100%	0.227	0.099	0.519	0.000

**Tidal water**				
no	1			
yes	0.232	0.101	0.533	0.001

**Deep water body** **(> 50 cm)**				
no	1			
yes	0.498	0.241	0.1.03	0.060

Increased water conductivity was associated with lower larval densities. Conductivity values above 2000 μS/cm lead to significant reductions (OR 0.458; 95% CI 0.212–0.990, p = 0.047), although this relationship was species dependent. Whilst *An. gambiae s.s. *and *An. arabiensis *were negatively affected by high salinity as estimated by conductivity (OR 0.206, 95% CI 0.080–0.531, p = 0.001), no association was found for *An. coustani s.l., An. melas *and *An. pharaoensis*. The pH varied very little between habitats (Table 2) and was not associated with larval density; neither was turbidity nor oxygen saturation.

### Emergence

A total of 106,234 insects were caught in the emergence traps in 715 trap-weeks. Dipterans represented 99% (105,204) of the catch, the remaining 1% were dragon- and damselflies (434) and waterbugs (596) (Table 6). Most Diptera were non-biting midges (Chironomidae, 85%), culicine mosquitoes represented 7.3% and anopheline mosquitoes 0.3%. Members of the *An. coustani *group were the dominant anopheline species (66%) caught. *An. gambiae s.l. *accounted only for 23% of the emerged anophelines and *An. pharaoensis *for 4% (13). The remaining 7% of anophelines could not be identified. After one week of storage in glycol under very hot conditions insect samples were often in bad condition when returned to the laboratory and therefore only 25 specimen of the *An. gambiae s.l. *sample could be successfully amplified for PCR analyses. Similar to the larval catches, *An. arabiensis *was the dominant species of the gambiae-complex (68%), followed by *An. gambiae s.s. *(20%) and *An. melas *(12%).

**Table 6:** Emergence fauna collected in 715 trap-weeks

**Taxa**	**Total**	**Average no./week/m**^2^
**Heteroptera**	596	1.06
**Odonata**	434	0.77
**Diptera**	105,204	187.20

Chironomidae	83,204	148.06
Simuliidae	1,518	2.70
Culicidae	7,514	13.37

Culicinae	7,201	12.81
Anophelinae	313	0.56

*Anopheles coustani s.l.*	207	0.37
*Anopheles gambiae s.l.*	73	0.13
*Anopheles pharoensis*	13	0.02
others	20	0.04

**Figure 4:**
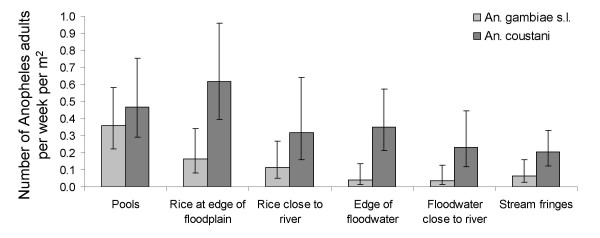
**Average adult anopheline densities per week and m^2 ^in different habitat types during the rainy season 2005**. Error bars show 95% confidence intervals.

Adult emergence occurred in all habitats but production differed between habitat types for both *An. gambiae s.l. *(p < 0.001) and *An. coustani s.l. *(p = 0.007). Average *An. gambiae *production was significantly higher in pools than all other habitats except in rice fields at the edge of the floodplain close to the upland. All other habitats were similarly productive (Table 7 and Figure 4). *An. coustani s.l. *emerged in higher densities than *An. gambiae s.l. *in all habitat types. *An. coustani s.l. *production was highest in rice fields at the edge of the floodplain close to the upland followed by pools (Table 7 and Figure 4).

**Table 7:** Mean adult *Anopheles *densities per habitat type and odds ratio in comparison to pools

	**Adult density/m**^2^			**Regression analyses**			
		
	Mean*	**95% CI**		**OR**	**95% CI**		**p**
***Anopheles gambiae s.l.***							
Pools	0.371	0.229	0.599	1.000			
Rice fields at edge of floodplain close to upland	0.170	0.082	0.352	0.459	0.192	1.096	0.080
Rice fields close to the river	0.118	0.050	0.277	0.317	0.119	0.846	0.022
Floodwater close to the edge of the floodplain	0.042	0.013	0.139	0.114	0.032	0.411	0.001
Floodwater close to the river	0.040	0.012	0.130	0.107	0.030	0.385	0.001
Stream fringes	0.066	0.027	0.165	0.179	0.064	0.502	0.001
							
***Anopheles coustani s.l.***							
Pools	0.483	0.301	0.777	1.000			
Rice fields at edge of floodplain close to upland	0.635	0.408	0.989	1.314	0.686	2.515	0.410
Rice fields close to the river	0.329	0.164	0.660	0.681	0.293	1.582	0.372
Floodwater close to the edge of the floodplain	0.360	0.219	0.592	0.745	0.374	1.481	0.401
Floodwater close to the river	0.238	0.123	0.461	0.493	0.218	1.112	0.088
							
Stream fringes	0.209	0.128	0.341	0.431	0.218	0.854	0.016

*GEE were used to calculate mean and 95% CI, with site ID as subject units, log linked mosquito

densities and habitat type as the factor

**Figure 5:**
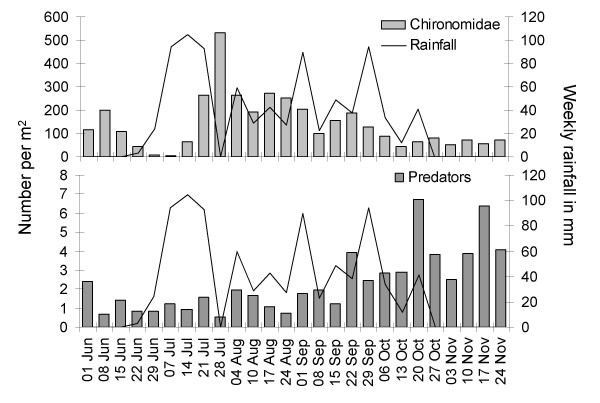
**Weekly emergence of chironomids and predatory insects per m^2 ^in relation to rainfall pattern**.

**Figure 6:**
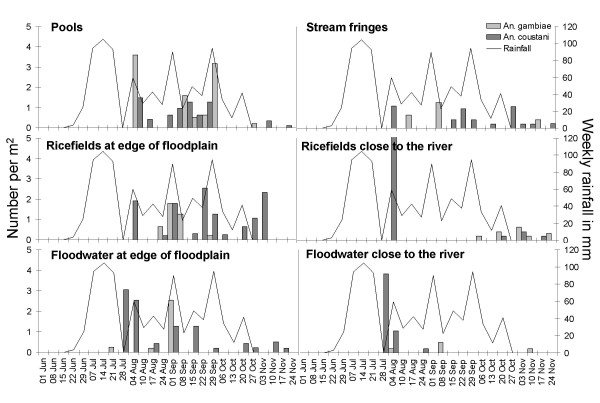
**Weekly emergence of *An. gambiae *and *An. coustani *per m^2 ^in relation to rainfall pattern**.

Emergence traps were placed in all habitat types from the beginning of June and rainfall commenced approximately 2 weeks later. Whereas other insects were caught throughout the sampling period (Figure 5) anopheline mosquitoes only started emerging from late July after the first peak in rainfall (Figure 6). Most adult anophelines emerged in August and September (83% of all *An. gambiae *and 68% of all *An. coustani*) when the highest larval densities were observed (Figure 3). The pools produced on average 0.74 (95% CI 0.31–1.16) *An. gambiae s.l. *and 1.06 (95% CI -0.04–2.15)*An. coustani s.l. *per m^2 ^per week during these two months. Stream fringes, the edge of floodwater and floodwater areas close to the river produced *An. gambiae *only on a few occasions. Rice fields showed a conspicuous seasonality depending on their location. Rice fields closer to the edge of the floodplains produced *An. gambiae *adults from the end of August till the end of September whereas none emerged at this time period in rice fields close to the river. In those, adults were only detected at the end of the rainy season in October/November.

**Figure 7:**
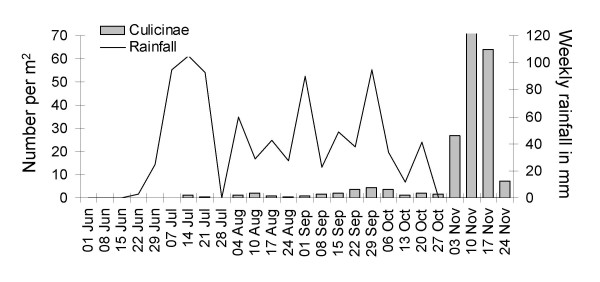
**Weekly emergence of culicine mosquitoes per m^2 ^in relation to rainfall pattern**.

Average culicine productivity was low in the beginning of the rainy season and only started to increase from September onwards with an increase of a magnitude in November after the rain had already stopped (Figure 7); 88% of all culicine mosquitoes were collected from rice fields at the edge of the floodplain close to the upland and 7% from the pools, the remaining 5% were collected from floodwater areas and rice fields close to the river.

All habitat types surveyed for insect emergence were similar in pH (6.7; 95% CI 6.5–6.8) and oxygen saturation (92%; 95% CI 80–104%). The average water depth under the floating emergence traps was 8 cm (95% CI 7–9 cm). Other abiotic and biotic characteristics varied significantly (Table 8). Pools, the habitat type with the highest *An. gambiae *production, were characterized by high average water temperature, very low water conductivity, high turbidity and increased presence of algae. This was the only habitat type that was not tidal. The insect community in these pools was characterized by the highest densities of predatory (carnivorous) organisms like dragon-and damselflies and beetles and high densities of chironomids which serve in their larval stage as food organisms for many fish and predatory invertebrates. Consequently, pools had the highest diversity index. In contrast, floodwater habitats and stream fringes had lower water temperatures and were characterized by high water conductivity, low turbidity and a poor insect diversity.

**Table 8:** Abiotic and biotic characteristics of six habitat types surveyed for insect emergence (values represent means (95% CI) of all sampling events during the rainy season 2005)

	Water temperature (°C)	Conductivity (μS/cm)	Turbidity (NTU)	Chironomida/m^2^/week	Odonata & Coleoptera/m^2^/week	Diversity Index	Frequency of algae present in habitat (%)
Pools	33.2 (31.1–35.5)^c^	323 (227–874)^a^	204 (97–310)^a,b^	166 (125–212)^a,b^	1.18 (0.99–1.38)^a^	0.5 (0.44–0.56)^a^	69.9
Rice close to upland	28.9 (25.8–32.0)^a,b^	3138 (1990–4286)^b^	316 (133–500)^a^	334 (231–439)^a^	1.11 (0.90–1.32)^a^	0.36 (0.31–0.42)^b^	61.6
Rice far from upland	30.0 (28.0–32.0)^b,c^	2502 (915–4088)^a,b^	206 (108–305)^a,b^	58 (38–79)^c^	0.32 (0.21–0.44)^b^	0.40 (0.35–0.44)^a,b^	18.5
Edge floodwater	27.6 (25.9–29.4)^a,b^	2064 (1608–2519)^a,b^	76 (40–111)^b^	105 (75–134)^b,c^	0.31 (0.20–0.41)^b^	0.32 (0.28–0.35)^b^	21.7
River floodwater	28.7 (27.1–30.3)^a,b^	1472 (926–2019)^a,b^	72 (38–105)^b^	59 (46–70)^c^	0.21 (0.09–0.32)^b^	0.35 (0.31–0.38)^b^	10.9
Stream fringes	25.7 (23.8–27.5)^a^	7199 (5618–8778)^c^	102 (59–144)^b^	149 (117–181)^a,b^	0.41 (0.31–0.51)^b^	0.33 (0.29–0.37)^b^	47.4
ANOVA/χ^2^	F(2,125) = 6.672, p < 0.001	F(5,119) = 20.65, p < 0.001	F(5,103) = 4.627, p = 0.001	F(5,709) = 12.735, p < 0.001	F(5,709) = 35.205, p < 0.001	F(5,709) = 8.090, p < 0.001	χ^2 ^= 151.1, df = 5, p < 0.001

Different letters indicate significance (p < 0.05) based on Gabriel post-hoc test for ANOVA, insect density was log transformed;

Chi^2 ^test was used for comparing the proportion of time algae were found in a habitat type

## Discussion

The identification of aquatic habitats preferably colonized by malaria vectors, and those most productive, remains a challenge since our results show that apparently similar habitats would not always contain anopheline larvae. Only two factors increased the probability of finding anopheline larvae in a habitat; namely the presence of tufts of grass or other short emergent vegetation and the presence of algae. Both factors have been identified to be of importance in a number of other studies throughout Africa [30-34]. The presence of algae in anopheline production remains controversial. Whilst algae can provide a viable food source for *Anopheles *larvae [32], algae are frequently associated with older habitats which may be less suitable for *Anopheles *development [30].

The density of larvae was dependent on a number of physical characteristics. Average larval densities decreased with increasing coverage of the water surface by tall vegetation, an observation that has also been made in various other environments throughout Africa [7,33,35] and was historically utilised for mosquito larval source management [7,36-39]. Larval density also decreased when the water was tidal which was the case for the majority of larger (>100 m perimeter) and deeper (>50 cm) habitats. These habitats were also characterized by a high water conductivity (>2000 μS/cm), which specifically reduced the larval density of the primary malaria vectors *An. gambiae s.s*. and *An. arabiensis*. These factors probably explain the observation made earlier in a longitudinal survey implemented under operational conditions [20] that larvae were more likely to be found in the first 1 km stretch from the edge of the floodplain than closer to the river.

*Anopheles arabiensis *was the most prevalent species of the *An. gambiae *complex in the study area, which is in agreement with previous findings from dry and humid savannah areas [40,41] but this is not reflected in the indoor collections from houses near the larval habitats, where *An. gambiae s.s. *dominates [42]. This might be due to the fact that *An. arabiensis *is a more exophilic mosquito and feeds to a large proportion on cattle which can be found closer to the habitats [43,44]. Nevertheless, the dominant *Anopheles *species in this survey was *An. coustani s.l.*, a mosquito that rests and bites outside [45]. *Anopheles coustani *and *An. pharaoensis *are considered secondary vectors of malaria in The Gambia but no published information is available about their role in malaria transmission in the country. Notably, *An. coustani *and *An. pharaoensis *represented a large proportion of the sampled anophelines; that is 44% of the identified larvae and 70% of adults. Recent studies from Senegal have shown that environmental changes can lead to changes in the vectorial system [41,46] and these may be further impacted if vector control strategies, which focus on indoor host-seeking females lead to a shift in species composition. In such events outdoor biting and resting mosquitoes may increase in importance as vectors of malaria [47].

Most primary and secondary adult malaria vectors emerged in August and September. The average *An. gambiae *production was significantly higher in confined pools than in all other habitats except in rice fields at the edge of the floodplain close to the upland, which share a number of characteristics with pools since they are more defined than other habitats, have high turbidity and more frequently contain algae. *Anopheles coustani *emerged in higher densities than *An. gambiae *in all habitat types surveyed. The overall productivity per m^2 ^of aquatic habitat in The Gambia was very low for vectors and other invertebrates alike. In relatively small and confined *An. gambiae *habitats in Kenya for example 18–200 larvae were sampled per area sampler (notably the area sampler was much smaller; 78.5 cm^2^), and 1–30 pupae collected per m^2 ^depending on habitat type and season [48]. In the western Kenyan highlands, a one year survey with emergence traps [49] estimated the average productivity of aquatic habitats to be 1.82 *An. gambiae *per m^2 ^per week; 14-times higher than the findings from The Gambia.

Confined habitats were most likely to have high densities of larvae and lead to the greatest number of adults emerging. They were typically characterized by freshwater with low conductivity, high turbidity, presence of algae and the lack of disturbance by tidal water movements. Vectors and other invertebrates alike thrived in this environment. This is probably a result of the protection that these habitats provide from changing environmental impact due to tidal water movement and the high nutrients present in these habitats. Freshwater streams ideally have a conductivity between 150–500 μS/cm to support a diverse aquatic life [50]. However, extremely high water conductivity due to high salinity and other dissolved ions, as measured in most habitats in this region of The Gambia, has a negative impact on primary production [51]. Run-off, plants, phyto- and zooplankton and other microorganisms are responsible for the provision of food for all primary consumers (e.g. chironomid and mosquito larvae), which serve as food sources for secondary consumers (e.g. predatory dragon- and damselfly larvae, bugs, fish). The vast floodwater area of this region of the Gambia River is poor in nutrients presenting little food for both, primary and secondary consumers. It is, therefore, not surprising that mosquito larvae were found associated with other invertebrates which are primarily predatory organisms. This extends also to the presence of fish in some habitats. In rice fields for example, the presence of small fish was positively associated with the density of vectors and other invertebrates (OR 2.883, 95% CI 1.141–7.284, p = 0.025). Invertebrate predators and small fish are natural enemies of mosquito larvae [52-54] and ovipositing mosquitoes are known to avoid habitats colonised by predators [55,56]. These apparently contradictory findings may indicate that greater reproductive success can be achieved by ovipositing females laying their eggs in water bodies with predators, where the chance of producing offspring is greater than that found in less salubrious water bodies, where there are no predators. Here water quality is perhaps more important than the presence of predators.

Two aspects affect larval distribution in aquatic habitats; the oviposition choice of a gravid female and the survival of larvae in the aquatic environment. Early instars of anopheline larvae were found in 88% of all habitats where larvae were present (cases), whilst late instars occurred only in 59% of sites. This result may indicate that oviposition occurs in a larger number of habitats perceived suitable by the ovipositing female but that the survival of larvae as expressed by larval density depends on factors associated with habitat size, stability and conductivity of water bodies. Another explanation for the wider distribution of early instars could be that the early instar larvae are more readily dispersed by the movement of tidal water than late instars. Binary analyses revealed that the presence of short vegetation and algae at the sampling point was associated with the presence of early, but not late instars, indicating that these factors, or their associated covariates like nutrients, bacteria and volatiles released from the water [57], are important for the adult female seeking oviposition sites, but that additional factors impact on larval survival.

Rice is the staple food for people in The Gambia and in this part of the country rice fields represent the majority of habitats. After the seasonal rains start in June, women plough their fields in the floodplains of the Gambia River and prepare raised beds for growing rice. Two sets of young plants are transplanted to the fields. The first are transplanted early on in the rainy season in paddies close to the landward edge of the floodplain in August. These are the first areas to flood after the soil becomes saturated following several heavy downpours. However, by October, most of them are drying out and the second set of young rice plants are transplanted to fields close to the river from late September to early October. Here the water is largely flowing in from the river. This explains the seasonality in adult emergence observed. Fields closer to the edge of the floodplains produced *An. gambiae *adults from the end of August until the end of September, whereas fields closer to the river produced adults at the end of the rainy season in October/November. The overall adult productivity over the season was similar in both fields far away and close to the river, which contradicts findings from a far more intensive but smaller study closer to Farafenni which found nearly all adults emerged from rice fields located within 100 m from the landward edge, away from the river (Jarju, L. personal communication). These contradictory findings suggest that within the smaller study area stronger tides (since it is nearer the river's mouth) may have reduced mosquito production close to the river. Distance from the edge of the floodplain was not a limiting factor either, demonstrating that adults are produced from within the flooded areas and not just on the landward fringe.

Over the entire sampling period, only 33 mosquito pupae were collected with area samplers. This confirms earlier observations [14] that pupae are rare, widely distributed and difficult to sample in the extensive habitats of The Gambia. The use of area samplers did not improve the sampling success in comparison to dipping and therefore cannot be used as a reliable tool to estimate emergence. Generally, the difficulty in sampling vectors in the highly mobile environment in the floodplain of The Gambia, especially closer to the river, needs to be emphasized. The productivity in tidal habitats might have been underestimated in contrast to defined pools using emergence traps since larvae travel with water and might have not emerged under the trap. It is simply far easier to sample mosquitoes in small, defined habitats with higher density per surface area than in large and thinly populated habitats with constant water movement.

## Conclusion

In an extensive habitat survey of an area of 400 km^2^, irrigated rice fields presented the vast majority of aquatic habitats with a total surface area of 4,300 ha (43 km^2^), the landward edge of the floodwater accounted for approximately 1000 ha (10 km^2^) of aquatic habitats whilst in total all pools covered a surface area of 2 ha (0.02 km^2^) only, representing only 0.03% of the potential mosquito breeding sites [20]. Therefore, highly productive breeding sites, like confined pools, are small in surface area in this part of The Gambia, whereas those with low productivity are large and widely distributed, and can be found throughout the rainy season. The vast majority of potential breeding sites are floodwater areas including extensive swamp rice cultivations over the entire width of the floodplain. All habitat types are equally colonized and even though the majority of sites have low larval density resulting in a low emergence rate, these tidal floodwater areas including rice fields present the majority of surface water in The Gambia. Even though risk factors could be identified for the presence and density of larvae and productivity of habitats, they indicate that anti-larval interventions in this part of The Gambia cannot be targeted in space or time during the rainy season.

## Consent

Persons shown in the photographs have consented to publication.

## Competing interests

The authors declare that they have no competing interests.

## Authors' contributions

UF, SWL, WT and SM designed the study. HS was responsible for the implementation of the study in the laboratory and the field, assisted by SM and EvL. UF analysed the data and drafted the manuscript. All authors read and approved the final manuscript.
